# Fuzzy support vector machine: an efficient rule-based classification technique for microarrays

**DOI:** 10.1186/1471-2105-14-S13-S4

**Published:** 2013-10-01

**Authors:** Mohsen Hajiloo, Hamid R Rabiee, Mahdi Anooshahpour

**Affiliations:** 1 Department of Computer Engineering, Sharif University of Technology, Azadi Ave., Tehran, Iran; 2 Department of Computing Science, University of Alberta, Edmonton, Alberta, Canada

## Abstract

**Background:**

The abundance of gene expression microarray data has led to the development of machine learning algorithms applicable for tackling disease diagnosis, disease prognosis, and treatment selection problems. However, these algorithms often produce classifiers with weaknesses in terms of accuracy, robustness, and interpretability. This paper introduces fuzzy support vector machine which is a learning algorithm based on combination of fuzzy classifiers and kernel machines for microarray classification.

**Results:**

Experimental results on public leukemia, prostate, and colon cancer datasets show that fuzzy support vector machine applied in combination with filter or wrapper feature selection methods develops a robust model with higher accuracy than the conventional microarray classification models such as support vector machine, artificial neural network, decision trees, k nearest neighbors, and diagonal linear discriminant analysis. Furthermore, the interpretable rule-base inferred from fuzzy support vector machine helps extracting biological knowledge from microarray data.

**Conclusions:**

Fuzzy support vector machine as a new classification model with high generalization power, robustness, and good interpretability seems to be a promising tool for gene expression microarray classification.

## Background

### Gene expression microarrays

Almost every cell of the body contains a full set of chromosomes and the same set of genes. But each cell finds its unique properties at a specific time by the fraction of genes which are turned on (expressed) in it. In each cell, transcription of the genetic information contained within the DNA into messenger RNA (mRNA) molecules is called gene expression. These mRNAs are later translated into the proteins that perform most of the critical functions of cells. Gene expression is a complicated process that allows a cell to respond both to environmental needs and to its own changing needs [1]. Upon completion of the Human Genome Project, human genome was sequenced and genes within the genome were identified [2]. Moreover, tools such as microarrays enabled scientists to study changes in expression level of a large set of genes simultaneously. These studies can lead to easier diagnosis or prognosis of complex diseases such as cancer and discovery of better drugs.

As it is almost impossible to study gene expression of a large number of genes provided by microarrays with traditional techniques, scientists use novel techniques such as machine learning algorithms to study microarrays. Microarray classification is a challenging task because: 1) there are a large number of genes and a small number of samples in microarray experiments and this high dimensionality leads to overfitted models, 2) various uncertainties and noises are associated with microarray experiments, and 3) biomedical community usually prefers biologically relevant models like rule-based models that can reveal interconnection of genes and black-box models with high generalization power are not necessarily the favorite options in this context [3]. Many machine learning algorithms are designed and applied for microarray classification problem which some of them provided fruitful results but due to the challenges of dealing with microarrays researchers are still working on designing more accurate, robust, and interpretable models for microarray classification.

### Microarray classification

Since microarrays have a large set of features but a small set of samples, feature selection methods are applied on them to eliminate noisy and irrelevant features. Furthermore, feature selection decreases the chance of overfitting and increases the chance of producing more understandable results. Two broad categories of feature selection techniques are filter and wrapper methods [4]. Filter methods are also categorized into univariate and multivariate approaches. The univariate filter methods first assign a score calculated by a score metric to each gene and then sort genes according to their score. A set of top-ranked genes are selected as the output of these feature selection techniques. On the other hand, the multivariate filter methods like multiple hypothesis testing models [5,6] consider the interconnection between features and are slower than univariate approaches. Wrapper methods pick subsets of genes from the initial set of genes and test the quality of these subsets by building a classifier on each subset. The subset of genes which brings the highest accuracy is selected as the output of wrapper feature selection techniques. It is important to note that univariate filter methods are usually faster than the multivariate filter methods as they ignore interconnection of genes and the multivariate filter methods are usually faster than wrapper methods as they ignore interactions with the classifier, but wrapper methods and multivariate filter methods consider interconnection of genes and have the chance of finding the best subset that work together in determination of the class label of the given problem.

Supervised learning is a process in which the class labels of a set of instances are given and by applying a learning method, we build a classifier which can be used later in determining the class label of new instances. There are different ways of evaluating the performance of a classifier like using a separate test data, cross-validation, bootstrap sampling, and sub-sampling as surveyed and described in [7,8]. Lu and Han [3], Pirooznia, Yang, Yang, and Deng [9], and Dudoit, Fridlyand, and Speed [10] have surveyed supervised learning methods applied on microarrays. These studies emphasize that in microarray's domain simple robust rule-based models with high generalization ability and less sensitivity to noise are preferred. Various classification models have been applied on microarrays like support vector machine (SVM) [11,12], artificial neural network (ANN) [13], decision trees [10,14], k nearest neighbors (KNN) [10], diagonal linear discriminant analysis (DLDA) [10]. Here, we review these methods in order to better understand the requirements of a good classifier in the microarray's context.

SVM in a binary classification problem finds maximum margin classifier among many different classifiers that can separate instances of two classes. The points that lie closest to this max-margin hyperplane are called the support vectors. The hyperplane can be defined using these points alone and the classifier only makes use of these support vectors to classify test samples. In the case that data points are not linearly separable in the input space, one solution is to map the input space into a higher dimensional feature space called kernel space [15]. SVM was used for microarray classification by Mukherjee et al. [11] and Furey et al. [12]. According to these studies, SVM produces accurate classifiers and is one the best classifiers used in microarray's domain. The reasons of its good performance are its ability in dealing with high dimensional problems, ability in dealing with noisy features, scalability, and its high generalization power.

ANN is an interconnected group of nodes that uses a computational model for information processing. It changes its structure based on external or internal information that flows through the network. ANN can be used to model a complex relationship between inputs and outputs and find patterns in data. This tool has been applied for microarray classification by Khan, Wei, and Ringnér [13] and showed good results in comparison with the other methods; however as it performs classification in a black box manner, it does not provide any insight on how the genes are correlated or which set of genes is more effective for classification.

A decision tree like classification and regression trees (CART) [16] consists of a set of internal nodes and leaf nodes. The internal nodes are associated with a splitting criterion which consists of a splitting attribute and one or more splitting predicates defined on this attribute. The leaf nodes are labelled with a single class label. The construction of the decision tree is usually a two-phase process. In the first phase, the growing phase, an overgrown decision tree is built from the training data. The splitting criterion at each internal node is chosen to split the data sets into subsets that have better class separability, thus minimizing the misclassification error. In the second phase, the pruning phase, the tree is pruned using some heuristics to avoid overfitting. Decision trees have been widely used in microarray studies. They have many advantages that make them a suitable choice for microarray classification like scalability, fast setup, independence of input parameters, and generating rule-based models. Disadvantage of decision trees that appears in high dimensional problems like microarray classification is that they become overfitted easily [10,14]. One way to overcome this limitation of decision trees is to build an ensemble model over them like the approach used in Li et al. [17].

In KNN classifier, class label of an unseen instance is determined by a majority voting on the class label of its k nearest neighbors and correlation or Euclidean distance is usually used to calculate distance of two samples. KNN was used for the purpose of microarray classification by Dudoit, Fridlyand, and Speed [10]. They have shown that nearest neighbor as a simple similarity based method has low error rate in microarray classification as it is not so sensitive to noisy samples but still has the drawback of working in a black-box manner.

LDA has been one of the most popular methods used in classification problems. The basic idea of LDA is to project high-dimensional data onto a low-dimensional space such that the data points are reshaped to maximize the class separability. The optimal projection of classical LDA is obtained by maximizing the between-class distance while minimizing the within-class distance [18]. In high dimensional problems with few samples like the case of microarrays a great challenge to classical LDA is the singularity and instability of the within-group sum of squares matrix or the sample covariance matrix. Therefore, the covariance matrix will be singular if the total number of observations in the training data (*N*) is less than the number of features (*P*) or might be very unstable and noisy if *N *is not significantly larger than *P*. It has been shown that the performance of LDA in high-dimensional situations is far from optimal. Variants of LDA are used for pattern classification like diagonal LDA (DLDA). DLDA is the same as LDA except that the covariance matrices are assumed to be diagonal. The DLDA algorithm has shown a good performance on gene expression microarray data [10]. Unlike classical LDA, DLDA does not explicitly require the number of features to be less than the size of the training set in theory.

But, as more features are added to a given model, the complexity of the model is increased and as a consequence the overfitting chance and the misclassification rate of new samples increases [19].

### Study motivation

Our goal is to develop an accurate and robust microarray classifier which outputs a useful rule-base suitable for information extraction as well. To do so we apply an additive fuzzy system to kernel machines, and demonstrate that, under a general assumption on membership functions, an additive fuzzy rule-based classification system can be constructed directly from the given training samples using the support vector learning approach.

## Methods

Here, we explain the feature selection methods, the supervised learning method, and the model evaluation strategy used in this study.

### Feature selection

The first step in microarray classification is to apply a feature selection method on microarray data to get rid of irrelevant genes and reduce the dimension of feature space. Here, we use a common filter feature selection technique (signal to noise ratio (SNR) [1]) and a common wrapper feature selection technique (support vector machine-recursive feature elimination (SVM-RFE)) [20,21].

SNR was used in the first reported study of application of machine learning in microarray classification domain conducted by Golub et al. [1] and was used in many microarray studies afterwards. In this filter method for each gene G_i _a score SNR_i _is calculated by (1). Then genes are sorted according to their SNR scores and a set of top ranked genes are selected as the output. In this formula, µ_i(+) _and σ_i(+) _respectively represent mean and standard deviation of positively labeled samples and µ_i(-) _and σ_i(-) _respectively represent mean and standard deviation of negatively labeled samples.

(1) $$\text{SN}{\text{R}}_{\text{i}}=\left|\frac{\mu_{i}\left(+\right)-\mu_{i}\left(-\right)}{\sigma_{i}\left(+\right)+\sigma_{i}\left(-\right)}\right|$$

SVM-RFE is a backward feature ranking wrapper feature selection method which considers interconnection of genes. SVM-RFE builds a support vector machine model on the whole set of genes and then eliminates one gene which its squared weight computed by optimizer is the smallest. Elimination of genes continues in this way until we get a group of genes with the highest classification power.

### Supervised learning

The second step in microarray classification is taken in order to build a reliable model on training samples which can correctly predict class label of test samples. Here, we apply fuzzy support vector machine algorithm for supervised learning [22,23]. This method has combined the good generalization performance and ability to work in high dimensional spaces of support vector machine algorithm with high interpretability of fuzzy rule based models. A model with these properties is expected to overcome the challenges of dealing with microarrays very well.

Consider a fuzzy model with m rules which each rule's antecedent is a conjunction of n terms. Each fuzzy rule is of the form:

(2) $$\text{Rule j}:\text{IF }{{\text{A}}_{\text{j}}}^{1}\text{ AND }{{\text{A}}_{\text{j}}}^{2}\text{ AND }\ldots \text{ AND }{{\text{A}}_{\text{j}}}^{\text{n}}\text{ THEN }{\text{b}}_{\text{j}}$$

Where b_j _∈ R and A_j_^k ^is a fuzzy set with membership function a_j_^k^: R→ [0,1] and j = 1,..., m and k = 1,..., n. If we choose product as the fuzzy conjunction operator, addition for fuzzy rule aggregation, and Center of Area (COA) rule for defuzzification, then the model becomes a special form of the Takagi-Sugeno (TS) fuzzy model [24] and the input-output mapping F: R^n^→ R of the model is defined as:

(3) $$F\left(\vec{x}\right)=\frac{\overset{m}{\underset{j=1}{\sum }}b_{j}{\text{Π}}_{k=1}^{n}a_{j}^{k}\left(x_{k}\right)}{\overset{m}{\underset{j=1}{\sum }}{\text{Π}}_{k=1}^{n}a_{j}^{k}\left(x_{k}\right)}$$

Where $\vec{x}={\left[x_{1},\ldots ,x_{\text{n}}\right]}^{\text{T}}$ is the input. Note that if the input space is not wholly covered by fuzzy rules, (3) will not be well-defined. To fix this problem a Rule 0 can be added to the rule base:

(4) $$\text{Rule 0}:\text{IF }{{\text{A}}_{\text{0}}}^{1}\text{ AND }{{\text{A}}_{\text{0}}}^{2}\text{ AND }\ldots \text{ AND }{{\text{A}}_{\text{0}}}^{\text{n}}\text{ THEN }{\text{b}}_{\text{0}}$$

Where b_0 _∈ R and a_0_^k^(*x_k_*) = 1, for k = 1, ..., n and any *x_k _*∈ R. Consequently the input-output mapping becomes:

(5) $$F\left(\vec{x}\right)=\frac{b_{0}+\overset{m}{\underset{j=1}{\sum }}b_{j}{\text{Π}}_{k=1}^{n}a_{j}^{k}\left(x_{k}\right)}{1+\overset{m}{\underset{j=1}{\sum }}{\text{Π}}_{k=1}^{n}a_{j}^{k}\left(x_{k}\right)}$$

In a binary classification task, $\text{sign}\left(F\left(\vec{x}\right)\right)$ shows class label of each input $\vec{x}$ and since the denominator of equation (5) is always positive class label of each input is computable by:

(6) $$Label\left(\vec{x}\right)=sign\left(b_{0}+\overset{m}{\underset{j=1}{\sum }}b_{j}\overset{n}{\underset{k=1}{\prod }}a_{j}^{k}\left(x_{k}\right)\right)$$

The membership functions for a binary fuzzy classifier defined above could be any function from R to 0[1]. In fuzzy support vector machine approach, functions are narrowed to a class of membership functions, which are generated from location transformation of reference functions [25], and the classifiers defined on them. A membership function µ is a reference function if and only if µ(x) = µ(-x) and µ(0) = 1. A reference function with location transformation has the following property for some location parameter $z_{j}^{k}\in R:$

(7) $$a_{j}^{k}\left(x_{k}\right)=a^{k}\left(x_{k}-z_{j}^{k}\right)$$

A translation invariant kernel K is given by:

(8) $$K\left(\vec{x},\vec{z_{J}}\right)=\overset{n}{\underset{k=1}{\prod }}a^{k}\left(x_{k}-z_{j}^{k}\right)$$

One particular kind of kernel, Mercer kernel, has received considerable attention in the machine learning literature [26] because it is an efficient way of extending linear learning machines to nonlinear ones. Mercer kernels have positive semi- definite matrices. A translation invariant kernel is a mercer kernel if and only if its Fourier transform is nonnegative. A translation invariant kernel (8) over reference functions with location transformation (7) is proved to be a Mercer kernel because of having nonnegative Fourier transform. A list of such reference functions with their Fourier transform is presented in table 1.

**Table 1:** 

	Reference Function	Fourier Transform
**Symmetric Triangle**	*μ*(*x*) = max(1 - *d*|*x*|,0), *d *> 0	$\text{F}\left[\mu \right]\left(\omega \right)=\frac{1}{\sqrt{2\pi }}\frac{Sin^{2}\left(\frac{\omega }{2d}\right)}{{\left(\frac{\omega }{2d}\right)}^{2}}$

**Gaussian**	$\mu \left(x\right)=e^{-dx^{2}},d>0,d>0$	$\text{F}\left[\mu \right]\left(\omega \right)=\frac{1}{\sqrt{2d}}e^{-{\frac{\omega }{4d}}^{2}}$

**Cauchy**	$\mu \left(x\right)=\frac{1}{1+\text{d}{\text{x}}^{2}},d>0$	$\text{F}\left[\mu \right]\left(\omega \right)=\sqrt{\frac{\pi }{2d}}e^{-\frac{\left|\omega \right|}{\sqrt{d}}}$

**Laplace**	*μ*(*x*) = *e*^-*d*|*x*|^, *d *> 0, *d *> 0	$\text{F}\left[\mu \right]\left(\omega \right)=\sqrt{\frac{2}{\pi }}\frac{d}{d^{2}+\omega^{2}}$

**Hyperbolic Secant**	$\mu \left(x\right)=\frac{2}{{\text{e}}^{\text{dx}}+{\text{e}}^{-\text{dx}}},d>0$	$\text{F}\left[\mu \right]\left(\omega \right)=\frac{1}{d}\sqrt{\frac{\pi }{2}}\frac{2}{{\text{e}}^{\frac{\pi \omega }{2\text{d}}}+{\text{e}}^{-\frac{\pi \omega }{2\text{d}}}}$

**Squared Sinc**	$\mu \left(x\right)=\frac{Sin^{2}\left(dx\right)}{d^{2}x^{2}},d>0$	$\text{F}\left[\mu \right]\left(\omega \right)=\text{max}\left(\sqrt{\frac{\pi }{2}}\left(\frac{1}{d}-\frac{\left|\omega \right|}{2d^{2}}\right),0\right)$

Fuzzy support vector machine is a fuzzy rule-based model in which membership functions are reference functions with location transformation and given input $\vec{x}$ determines output class label by equation (9) in which $K\left(\vec{x},\vec{z_{J}}\right)$ is a Mercer kernel defined by equation (8).

(9) $$Label\left(\vec{x}\right)=sign\left(b_{0}+\sum_{j=1}^{m}b_{j}K\left(\vec{x},\vec{z_{J}}\right)\right)$$

Given a supervised learning problem in which training input is described by $\left(\vec{x},\vec{y}\right)$

where $\vec{x}=\left[\vec{x_{1}},\ldots ,\vec{x_{p}}\right]$ and $\vec{y}=\left[y_{1},\ldots ,y_{p}\right]$ and having a Mercer kernel in hand, kernelized support vector machine tries to optimize the following:

$$\text{Maximize W}\left(\vec{\alpha }\right)=\sum_{i=1}^{l}\alpha_{i}-\frac{1}{2}\sum_{i,j=1}^{l}\alpha_{i}\alpha_{j}y_{i}y_{j}K\left(\vec{x_{\iota }},\vec{x_{J}}\right)$$

$$\text{Subject to C}\geq \alpha_{i}\geq 0,\text{i}=1,\ldots l\text{ and }\sum_{i=1}^{l}\alpha_{i}y_{i}=0.$$

Solving (11) for $\vec{\alpha }$ gives a decision function of the form:

$$f\left(\vec{x}\right)=sign\left(b_{0}+\sum_{j=1}^{m}\alpha_{j}y_{j}K\left(\vec{x},\vec{x_{J}}\right)\right)$$

**Figure 1:**
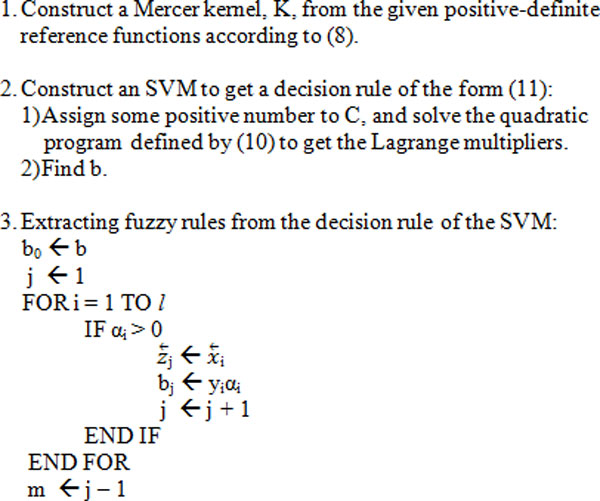
**Rule extraction algorithm from fuzzy support vector machine**.

Comparison of equation (9) and (11) shows how support vector machine and fuzzy rule based systems are related to each other. Given reference functions *a^k^*(*x_k_*), (k = 1,...,n) associated with n input variables and set of training samples $\left(\vec{x_{\iota }},\vec{x_{J}}\right)$. Steps of setting up fuzzy support vector machine classification model and rule extraction are as shown in Figure 1.

### Evaluation strategy

In order to assess the performance of the proposed model and compare the accuracy of this classifier with the accuracy of the state-of-the-art classifiers, 10-fold cross validation strategy is used.

## Results

### Datasets

The proposed methods have been evaluated by three publicly available microarray datasets, which are leukemia dataset [1], prostate cancer dataset [27], and colon cancer dataset [28]. The following is a brief introduction about these datasets, while more detailed information can be found from, respectively, the indicated data resources.

**Leukemia dataset: **this dataset was originally used by Golub et al. [1]. It contains the expression levels of 6817 gene probes of 72 patients, among which, 47 patients suffer from the acute lymphoblastic leukemia (ALL) and 25 patients suffer from the acute myeloid leukemia (AML).

**Prostate cancer dataset: **this microarray dataset is originally provided by Singh et al. [27]. The dataset *A *is used in the experiments in this paper. The dataset contains 102 samples, 52 are prostate tumor samples and 50 normal samples, and each array has 12,600 gene probes.

**Colon cancer dataset: **this microarray dataset was first used by Alon et al. [28]. The dataset contains 62 samples, 40 colon cancer cases and 22 healthy controls, and each array has 2000 gene probes.

### Hypotheses and experimental design

In this section we review the four hypotheses that we defined to explore power of fuzzy support vector machine model in microarray studies.

**Hypothesis 1: **how is the performance of fuzzy support vector machine model in comparison with the performance of common classification models used on microarrays like SVM, ANN, decision trees like CART, KNN, and DLDA?

**Experiment 1: **we train all these classifiers on the original microarray datasets without doing any kind of feature selection and compare the accuracy of these models.

**Experiment 2: **we apply SNR method for gene selection and then we train all these classifiers and compare test accuracy of these models.

**Hypothesis 2: **does fuzzy support vector machine model benefit from feature selection?

**Experiment 3: **we first set up fuzzy support vector machine classifier without taking the feature selection step and then we set up fuzzy support vector machine classifier with taking the feature selection step by use of both SNR and SVM-RFE methods and then compare the accuracy of these models.

**Hypothesis 3: **is the output rule-base generated by fuzzy support vector machine model useful for extracting meaningful information for biomedical researchers?

**Experiment 4: **as an instance of the rule-base generated by fuzzy support vector machine, we first apply SNR feature selection method on leukemia dataset and select three genes and then we set up fuzzy support vector machine classifier on those genes. We explore some characteristics of the model by taking a deeper look at the rule-base.

**Hypothesis 4: **is fuzzy support vector machine model a robust one for microarray classification?

We have two parameters in fuzzy support vector machine classification model: (1) C which is the misclassification cost of support vector machine classifier and (2) the number of selected features. In order to explore the sensitivity of the model to these parameters we have designed two experiments.

**Experiment 5: **we use SNR gene selection method to select 3 genes and after that we set up fuzzy support vector machine models on these genes but by changing the value of C parameter in fuzzy support vector machine and we capture accuracy of the models. We have changed the value of C between 1, 10, 100, 1000, and 10000.

**Experiment 6: **we select features by applying both SNR and SVM-RFE feature selection methods and after that we set up fuzzy support vector machine models on selected features. We repeat this experiment by selecting different number of features. The number of selected features is 2, 3, 5, 7, 10, 15, 30, 50, 100, and 150.

## Results and discussions

**Experiment 1: **the result of running the first experiment is presented in table 2. Fuzzy support vector machine classifier is sensitive to noisy and irrelevant features more than SVM, KNN, and DLDA classifiers but is not as sensitive as ANN and CART classifiers.

**Table 2:** Accuracy of fuzzy support vector machine model versus accuracy of common classification models used on microarrays when no feature selection step is taken.

	Leukemia	Prostate Cancer	Colon Cancer
**Fuzzy Support Vector Machine (FSVM)**	90.18 %	91.18 %	77.42%

**Support Vector Machine (SVM)**	94.36 %	93.55 %	80.70%

**Artificial Neural Network (ANN)**	76.81 %	81.09 %	75.80%

**Decision Tree (CART)**	69.63 %	73 %	69.35%

**K Nearest Neighbor (KNN, K = 3)**	94.18 %	93.27 %	72.58%

**Diagonal Linear Discriminant Analysis (DLDA)**	95.27 %	94.27 %	75.80%

**Experiment 2: **the result of running the second experiment is presented in table 3. As you see in this table the performance of fuzzy support vector machine classifier is better than SVM, ANN, KNN, CART, and DLDA classifiers when we use a feature selection method as simple as SNR before setting up the classification model.

**Table 3:** Accuracy of fuzzy support vector machine model versus accuracy of common classification models used on microarrays when SNR is used for feature selection

	Leukemia	Prostate Cancer	Colon Cancer
**Fuzzy Support Vector Machine (FSVM)**	98.57 %	95.18 %	93.75%

**Support Vector Machine (SVM)**	97.27 %	93.63 %	90.03%

**Artificial Neural Network (ANN)**	94.54 %	94.27 %	87.10%

**Decision Tree (CART)**	91.81 %	89.09 %	83.87%

**K Nearest Neighbor (KNN, K = 3)**	96.36 %	95.18 %	87.10%

**Diagonal Linear Discriminant Analysis (DLDA)**	96.18 %	94 %	88.71%

**Experiment 3: **the result of running the third experiment is shown in table 4. As you see in this table the performance of fuzzy support vector machine model when SNR or SVM-RFE feature selection methods applied is better than the performance of fuzzy support vector machine model in case no feature selection step is taken.

**Table 4:** Comparison of performance of fuzzy support vector machine model with and without taking feature selection step

	Leukemia	Prostate Cancer	Colon Cancer
**Fuzzy Support Vector Machine without Feature Selection**	90.18 %	91.18 %	77.42%

**Fuzzy Support Vector Machine with SNR**	98.57 %	95.18 %	93.75%

**Fuzzy Support Vector Machine with SVM-RFE**	98.75 %	94.27 %	96.77%

**Figure 2:**
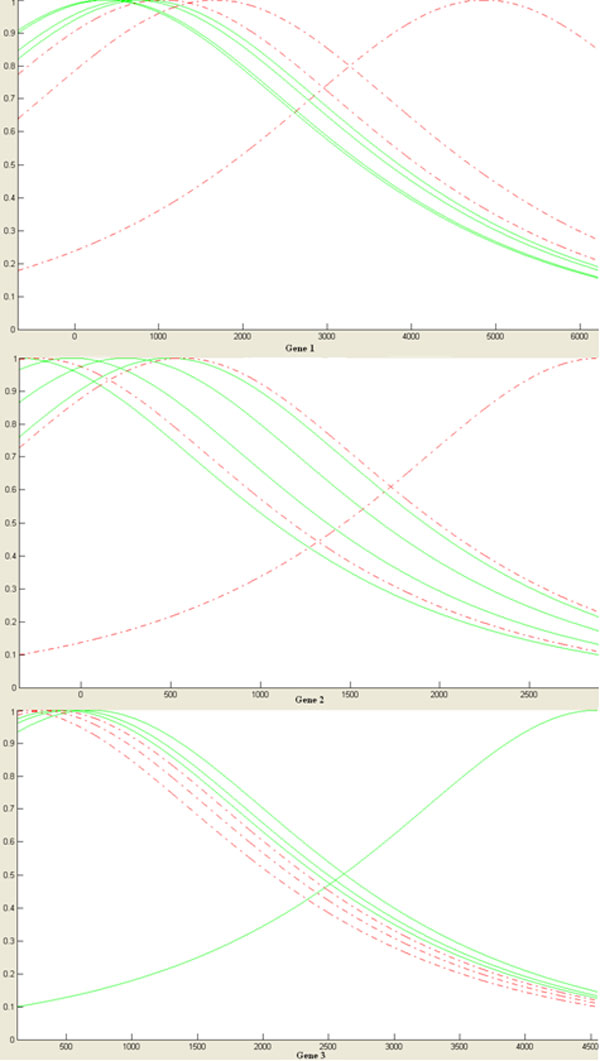
**Rule-base of fuzzy support vector machine on leukemia dataset**.

**Experiment 4: **the result of running the forth experiment is presented in table 5 and 6 and Figure 2. This rule-base has seven rules and each rule has three conjunctions in antecedent part and a consequent. A positive consequent means that rule is suitable for detecting positively labeled samples and a negative consequent means that rule is suitable for detecting negatively labeled samples. Due to the application domain of microarrays, rule-base models are of greater interest for biologists. We observe the followings in the rule-base generated by fuzzy support vector machine model:

**Table 5:** Rule-base of fuzzy support vector machine on leukemia dataset

	Gene 1	Gene 2	Gene 3	Consequent
**Rule 1**	408	252	474	1.946602
**Rule 2**	360	493	686	8.777722
**Rule 3**	827	-345	4555	0.703419
**Rule 4**	700	-49	553	5.852918
**Rule 5**	1050	565	389	-13.063
**Rule 6**	4863	2892	126	-0.44611
**Rule 7**	1671	-245	275	-3.77159

**Table 6:** Specification of genes of rule-base of fuzzy support vector machine on leukemia dataset

	Gene Name	Min Expression Level	Max Expression Level
**Gene 1**	Zyxine	-674	6218

**Gene 2**	PCF	-345	2892

**Gene 3**	TCF3	126	4555

• This rule-base contains seven rules. Therefore, only seven samples have a Lagrange multiplier other than zero when support vector machine optimizer is applied.

• This rule-base has three parts as antecedent. That is because the model is trained over three genes.

• The consequent of each rule shows the multiplication of the class label of the sample in the value of the Lagrange multiplier of the sample as mentioned in method section. This number can be used as the power of each rule. Rules with larger consequents are of greater importance and we expect to have greater coverage.

As a result, we can see that out of huge sized microarray with 6817 genes and 38 samples, we have created a model that decides class label of a test sample by just taking into account 3 of those genes and 7 of those samples with high accuracy. It is important to emphasize that fuzzy support vector machine is not the only interpretable model suggested for microarray classification and there are other interpretable models like the iGEC proposed by Ho, Hsieh, Chen, and Huang [29] for microarray classification.

**Figure 3:**
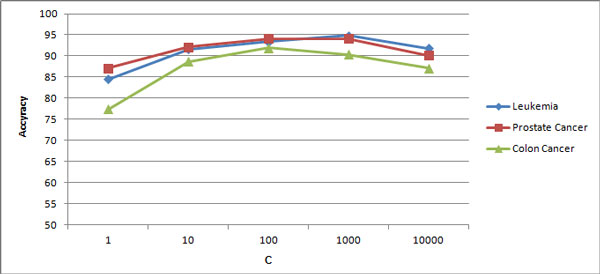
**Accuracy of fuzzy support vector machine for different values of C**.

**Experiment 5: **the result of running the fifth experiment is shown in Figure 3. As Figure 3 shows, the accuracy of the model leans toward to an optimum point when we increase the value of C from 1. But as you notice the fuzzy support vector machine model is not too much sensitive to the value of misclassification cost (C) parameter.

**Figure 4:**
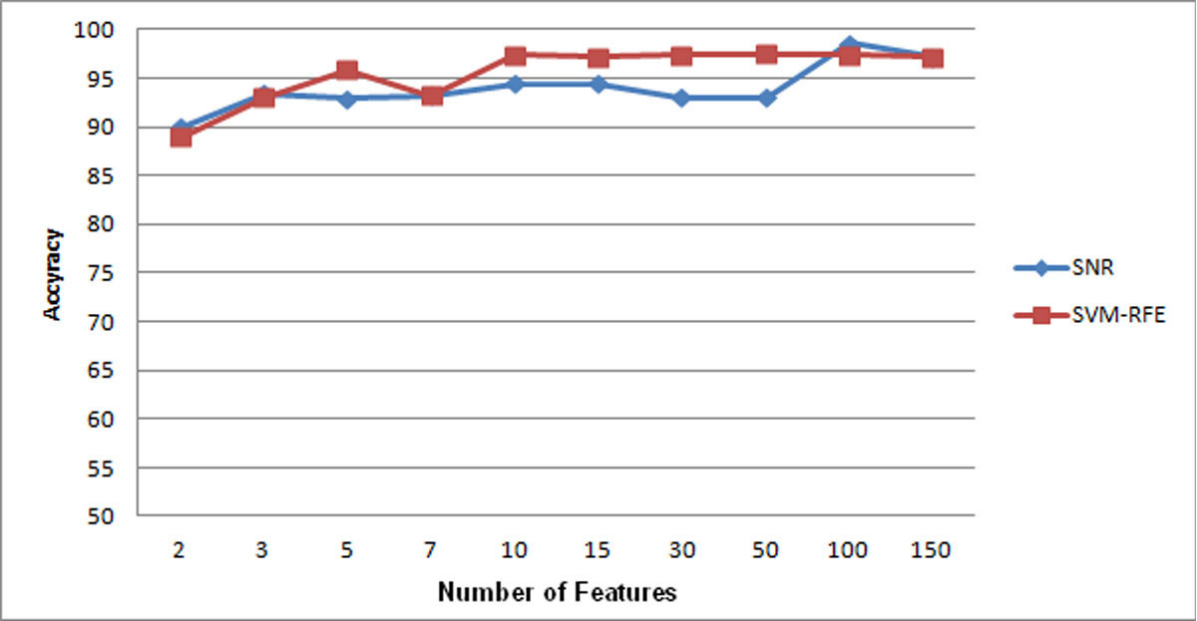
**Comparison of feature selection methods on leukemia dataset**.

**Figure 5:**
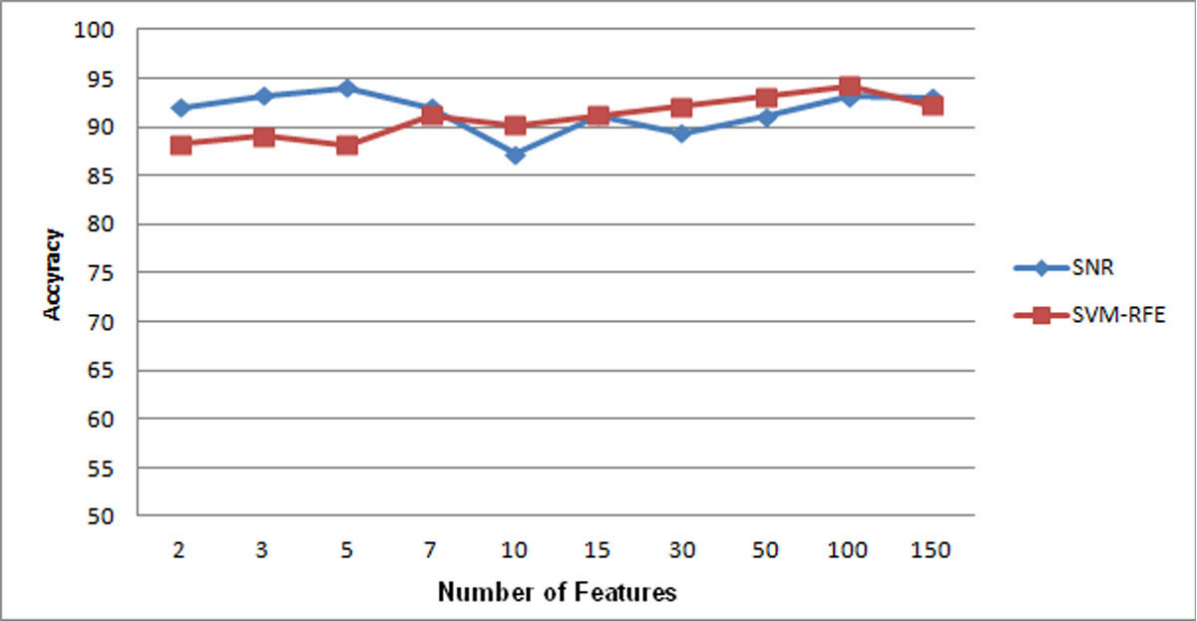
**Comparison of feature selection methods on prostate cancer dataset**.

**Experiment 6: **the result of running the sixth experiment is shown in Figure 4, 5, and 6. As you see, models built on features selected by both techniques are not sensitive to the changes of number of genes.

**Figure 6:**
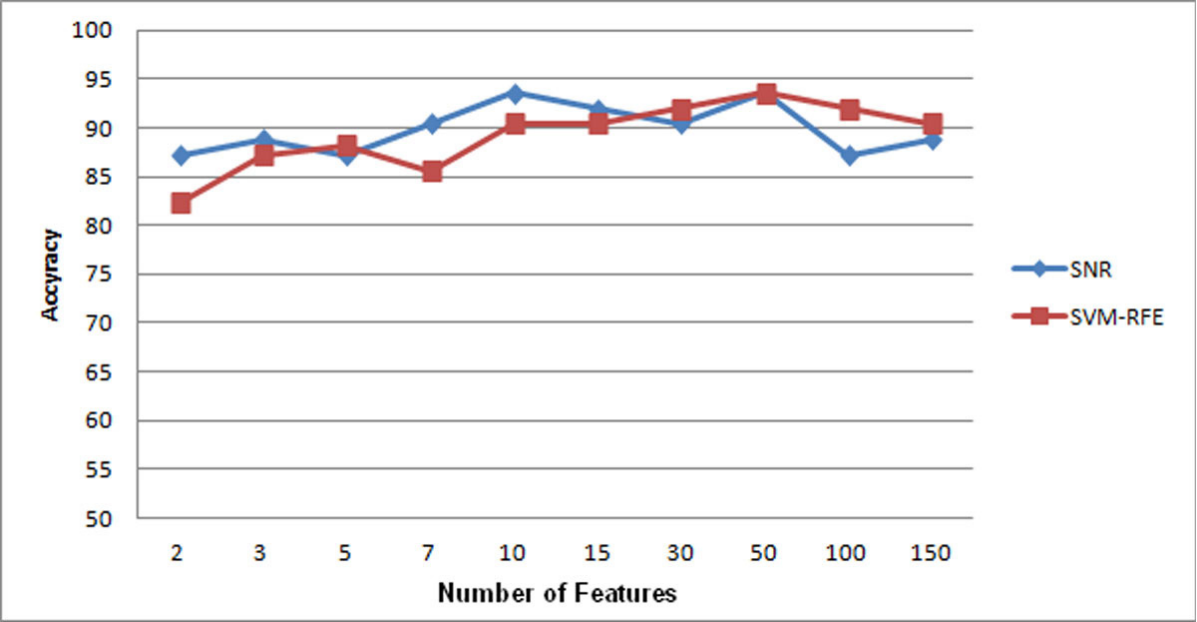
**Comparison of feature selection methods on colon cancer dataset**.

## Conclusions

In this paper, we have analyzed the prediction power of fuzzy support vector machine classifier on three different microarray classification problems. The experimental results show that fuzzy support vector machine has several advantages for microarray classification. First, fuzzy support vector machine combined with a feature selection method has higher generalization ability than common classification models for microarrays like support vector machine, artificial neural network, decision trees, k nearest neighbor, and diagonal linear discriminant analysis. Second, this model is robust in terms of the value of the parameter C and is not sensitive to the changes of the number of selected features. Third, the output rule-base of this model is a useful tool for extracting biological knowledge from microarray data. The authors believe that an accurate and interpretable model like fuzzy support vector machine finds high interest among biologist, physicians, and bioinformaticians who work on microarray classification problem.

## Competing interests

The authors declare that they have no competing interests.

## Authors' contributions

MH designed the experiments, participated in the implementation of the FSVM technique, and drafted the manuscript. HRR supervised the study and provided manuscript edits. MA implemented the FSVM technique and edited the manuscript. All authors read and approved the final version of the manuscript.

## Declarations

The publication costs for this article were shared by all authors.

This article has been published as part of BMC Bioinformatics Volume 14 Supplement 13, 2013: Selected articles from the 9th Annual Biotechnology and Bioinformatics Symposium (BIOT 2012). The full contents of the supplement are available online at http://www.biomedcentral.com/bmcbioinformatics/supplements/14/S13
